# News media coverage of euthanasia: a content analysis of Dutch national newspapers

**DOI:** 10.1186/1472-6939-14-11

**Published:** 2013-03-06

**Authors:** Judith AC Rietjens, Natasja JH Raijmakers, Pauline SC Kouwenhoven, Clive Seale, Ghislaine JMW van Thiel, Margo Trappenburg, Johannes JM van Delden, Agnes van der Heide

**Affiliations:** 1 Department of Public Health, Erasmus MC, PO Box 2040, Rotterdam, 3000 CA, the Netherlands; 2 Julius Center, University Medical Center Utrecht, the Netherlands Huispost Str 6.131, Utrecht, postbus 85500 3508 GA, the Netherlands; 3 Department of Sociology and Communications, Brunel University, Uxbridge, Middlesex, UB8 3PH, UK; 4 Utrecht School of Governance, University of Utrecht, Bijlhouwerstraat 6, Utrecht, 3511 ZC, the Netherlands

**Keywords:** Euthanasia, Media, Content analysis

## Abstract

**Background:**

The Netherlands is one of the few countries where euthanasia is legal under strict conditions. This study investigates whether Dutch newspaper articles use the term ‘euthanasia’ according to the legal definition and determines what arguments for and against euthanasia they contain.

**Methods:**

We did an electronic search of seven Dutch national newspapers between January 2009 and May 2010 and conducted a content analysis.

**Results:**

Of the 284 articles containing the term ‘euthanasia’, 24% referred to practices outside the scope of the law, mostly relating to the forgoing of life-prolonging treatments and assistance in suicide by others than physicians. Of the articles with euthanasia as the main topic, 36% described euthanasia in the context of a terminally ill patient, 24% for older persons, 16% for persons with dementia, and 9% for persons with a psychiatric disorder. The most frequent arguments for euthanasia included the importance of self-determination and the fact that euthanasia contributes to a good death. The most frequent arguments opposing euthanasia were that suffering should instead be alleviated by better care, that providing euthanasia can be disturbing, and that society should protect the vulnerable.

**Conclusions:**

Of the newspaper articles, 24% uses the term ‘euthanasia’ for practices that are outside the scope of the euthanasia law. Typically, the more unusual cases are discussed. This might lead to misunderstandings between citizens and physicians. Despite the Dutch legalisation of euthanasia, the debate about its acceptability and boundaries is ongoing and both sides of the debate are clearly represented.

## Background

The role of medicine and society in addressing the needs of patients who suffer unbearably and who request for their life to be ended is frequently debated. In the Netherlands, euthanasia was a topic of debate for many decades, eventually resulting in the legal regulation of euthanasia and physician-assisted suicide [1]. In several other countries, comparable regulation is in place or is currently being debated. These developments often yield emotional responses, and have given euthanasia and other life-ending practices, such as assisted suicide, a prominent place in news reporting.

Dutch euthanasia law defines euthanasia as the intentional ending of a life by the administration of medication by a physician at the explicit request of a patient [1], a definition that has been broadly accepted and adopted in legal regulations in other countries and in research. It is possible, however, that some people, amongst whom may be patients and healthcare providers, have different understandings of what euthanasia involves, potentially confusing them as to what is available under Dutch law, and certainly confusing the ethical debate. A recent Dutch study showed for example that several citizens considered the use of palliative sedation and the ending of life of severely ill newborns to be euthanasia [2].

Many people are exposed to media coverage of end-of-life care issues and mass media is a powerful source of information for the public [3]. Studies have shown, for example, that cancer news coverage influences the beliefs and behaviours of patients and healthcare providers [4,5]. It is, therefore, important to determine what practices are being referred to when newspaper reports label events as ‘euthanasia’, and to understand how news reporting represents arguments for or against the practice. Therefore, we studied reports about euthanasia in Dutch newspapers to investigate which practices the term ‘euthanasia’ refers to, and to study the content of these reports, in particular with respect to arguments for and against the use of euthanasia.

## Methods

We conducted a content analysis of articles from seven nationwide Dutch newspapers. We selected newspapers because these media have been shown to be a primary source of trusted health information [6]. Our selection strategy was aimed at obtaining a balance between newspapers with a large circulation and newspapers with frequent reports about euthanasia. We also strived for variance in readership profiles, especially with respect to the educational level and the religious disposition of the readers, this latter traditionally being an important factor in Dutch society, reflected in the editorial orientation of some newspapers. We selected, therefore, the following newspapers: *Nederlands Dagblad, Trouw, NRC, Volkskrant, AD, Telegraaf,* and *Spits!.* These newspapers are circulated in hard copy as well as electronically.

Articles were identified through LexisNexis, a database which contains 98% of all Dutch newspaper articles. Our search period was January 2009 until May 2010. We selected those articles that contained the word “euthanasia” at least twice. We included regular news reports, letters to the editor, comments and features.

**Figure 1:**
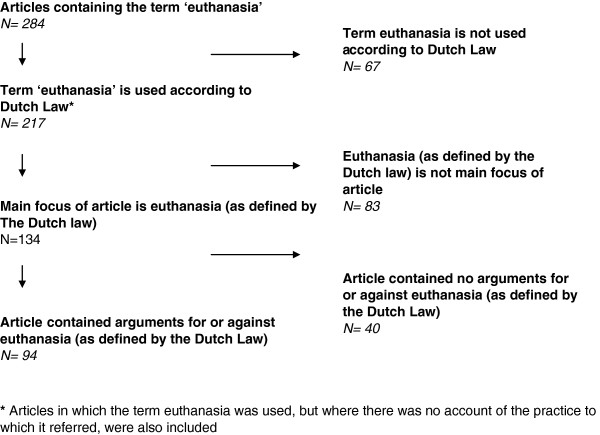
Flowchart of sampling decisions.

To ensure reliable coding, we developed, pretested and piloted a coding instrument, containing three stages. The first stage focussed on the use of the term euthanasia. Articles included in the sample were coded to determine whether the term euthanasia was used in accordance with the legal definition, and if not, to what other practice it referred. In the second stage, we first established whether euthanasia, according to the legal definition, was the main topic of the article or not. At this stage we also included articles in which the term euthanasia was used, but where there was no account of the practice to which it referred. The articles were coded to determine (1) whether they referred to specific patient groups, and if so, which; (2) whether the message of the article was supported by numbers (indicating a fact-based rather than purely opinion piece); and (3) whether specific organisations or associations were used as sources of information, and if so, which. Finally, in the third stage, we assessed whether these articles contained one or more arguments for or against euthanasia, and we listed and classified the content of the arguments. See Figure 1 for a flowchart of the inclusion and coding process.

Each article was scored by two researchers (JR and NR) independently. Subsequently, the codes were compared and discussed when different. There were few differences in the scoring, and consensus was always reached readily.

## Results

The search yielded 284 articles containing the term euthanasia at least twice (see Figure 1: Flowchart of sampling decisions).

In 60% of these articles (n = 170) the practice referred to by the term euthanasia was made clear (Table 1). In 24% of all articles (n = 67), the term euthanasia was used for practices outside the scope of Dutch euthanasia law, that is, not referring to the intentional ending of life by a physician at the explicit request of a patient. This concerned in most instances use of the term euthanasia for withholding or withdrawing potentially life-prolonging treatments (8% of all articles), for assisted suicide by physicians (2%) or by others (5%), and for active ending of life without an explicit request of the patient (4%, such as ending of life of newborns). More rarely, the term euthanasia referred to the killing of animals, to the cessation of eating and drinking by a patient, to suicide, or to the disproportionate increase of pain medication without a patient request. In three articles, the term euthanasia was used as a metaphor: e.g. ‘Facebook euthanasia’ (the deletion of friends on Facebook) and ‘workability euthanasia’ (firing employees). See Table 2 for some examples.

**Table 1:** Use of the term euthanasia in Dutch newspaper articles

	**n = 284 %**
The practice to which the term ‘euthanasia’ was applied was described	60 (n = 170)
The term euthanasia was used for practices outside the scope of the euthanasia Law	24 (n = 67)
*Where ‘euthanasia’ described practices outside the scope of Dutch euthanasia law it referred to:*	
Forgoing life prolonging treatments	8 (n = 24)
Assistance in suicide by non-physicians	5 (n = 13)
Active ending of life without request of the patient	4 (n = 12)
Assistance in suicide by physicians	2 (n = 6)
Killing of animals	1 (n = 3)
Metaphor	1 (n = 3)
Ceasing eating and drinking	1 (n = 2)
Suicide	1 (n = 2)
Disproportionate increase of pain medication	1 (n = 2)

**Table 2:** Examples of use of the term ‘euthanasia’ for practices outside the scope of the euthanasia Law

**Theme**	**Example**
Forgoing life prolonging treatments	The euthanasia of Eluana Englaro in Italy has led to much commotion. Premier Silvio Berlusconi tried two weeks ago at the very last minute to prevent the dehydration of the woman by submitting a provisional law. (De Telegraaf, 28-2-2009)
Active ending of life without request of the patient	Guidelines euthanasia babies under debate (Trouw, 3-12-2009)
Killing of animals	Almost two hundred chinchillas in the unique shelter of Vida Nueva in Musselkanaal will soon get an injection if thousands of dollars are not made available before the 23rd of July. This is desperately needed to provide for their care. Administrator Ellen Mulder is desperate: "Euthanasia is perhaps the most animal-friendly resolution when there is no other solution”. (De Telegraaf, 20-05-2009)
Metaphor	Minister Donner (of Social affairs and Employment) […] thinks it should be possible to pay older employees less allowances. This debate, however, evokes negative feelings. If you, as an employer, practice demotion, you practice -as it were- 'workability euthanasia'. (NRC Handelsblad, 10-12-2009)

In 134 of the 284 articles, euthanasia was the main topic. 68% of the 134 articles described the use of euthanasia for specific patient groups (Table 3). These concerned terminally ill patients in 36%, older persons in 24%, persons with dementia in 16%, persons with a psychiatric disorder in 9%, and persons in a coma in 5%. Of 134 articles, 37% were supported by the use of numbers derived from reports of scientific research or opinion polls. Several articles cited opinions or statements from particular organizations. These included political parties, government ministries, medical professional organizations, ‘Right to Die-NL’ (a pro-euthanasia organization) and advisory or executive organizations (e.g. Dignitas in Switzerland). Patient organizations and religious institutions were mentioned less often.

**Table 3:** Characteristics of the newspaper articles

	**N = 134 %**
*Persons*^*a*^	
Terminally ill persons	36
Older persons	24
Persons with dementia	16
Persons with a psychiatric disorder	9
Persons in a coma	5
Other persons	12
Not specified	32
*Message of the article is supported by numbers*	37
*Organisations or associations used as sources*^*a*^	
Political parties	22
‘Right to Die-NL’, advisory or executive organisations (Dignitas, Exit, De Einder)	20
Ministry/Public Prosecutor/Lower House	13
Medical professional associations	13
Support and Consultation for Euthanasia in the Netherlands (SCEN)^*b*^, Regional Review Committees for euthanasia	13
United Nations	11
Vatican, or other religious institutions	3
Patients’ or older persons’ associations	1
No organisations or associations cited	31

^a^More than one possibility per article.

^b^Professional organisation that can be consulted for advise about euthanasia, one of the procedural requirements for legal euthanasia in the Netherlands.

**Figure 2:**
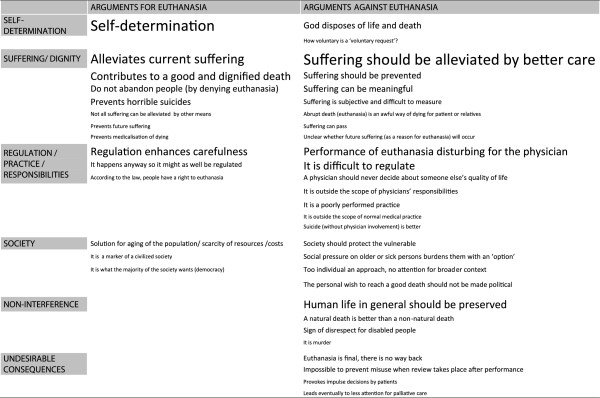
Arguments for and against euthanasia in the Dutch newspapers.

Finally, those articles that contained one or more arguments for or against euthanasia were scrutinized (94 of the 134 articles, 70%). See Figure 2 (Arguments for and against euthanasia in the Dutch newspapers) for a visual overview of the results, see Additional files 1 and 2 for a detailed account and see Tables 4 and 5 for some examples. Of these 94 articles, 67% contained at least one argument for euthanasia and 74% at least one argument against. Nearly half (45%) of these articles contained arguments both for and against. The number of arguments for euthanasia ranged from 0 to 5 (mean: 1.33) and the number of arguments against from 0 to 7 (mean 1.72).

**Table 4:** Examples of arguments for euthanasia in Dutch newspaper articles

**Theme**	**Example**
Self-determination, patient right	The Royal Dutch Medical Association thinks that "euthanasia is neither a right of the patient nor the duty of the physician”. This is wrong. euthanasia should be a patient's right. Yes, the desire to end life is subjective and therefore only the person him or herself can say whether it is the right thing. It is ridiculous that the government believes that we are too immature, too insignificant to make decisions about our lives ourselves. Shame. (letter, Trouw, 13-6-2009)
Contributes to a good and dignified death	Of course it is very emotional. I think that I did not sleep at all, each night following a euthanasia case. But on the other hand, the serenity, the stateliness almost that nearly always emerges when there is complete acceptance of death, is also very beautiful. What I learned from this is that dying is not terrible. Saying goodbye: that's terrible. (Trouw, 9-1-2010)
Regulation enhances carefulness	The Euthanasia Act, Anne-Mei The concludes, was passed in order to protect society against unwanted euthanasia practices. The benefit to the doctor is that he knows where he stands. (Trouw, 28-11-2009)
Solution for aging of the population/ scarcity of resources / medical costs	If there are no taboos any more for possible financial cuts, give everyone from e.g. 80 or 85 years the right to euthanasia. The advantages are clear, the government needs to pay less old-age pension, the pension funds will for obvious reasons also be happy, and health insurers do not need to spend fortunes to aim for a final age of 120. I think there are many older people who would make use of this. It is possible that this would yield more than 4 billion per year. So let the state pension age just remain at 65. (letter, de Volkskrant, 19-09-2009)

**Table 5:** Examples of arguments against euthanasia in Dutch newspaper articles

**Theme**	**Example**
Suffering can/should be alleviated by better care	Amyotrophic lateral sclerosis (ALS) is an incurable disease where adults increasingly have difficulties using their muscles. One in five ALS patients die by euthanasia in the Netherlands, while the overall euthanasia rate is only 1.8. […] Foreign doctors have found this rate unacceptably high and have questioned the quality of care for ALS patients in the Netherlands. Theay have also wondered whether Dutch doctors perhaps undertreat depression and related death wishes in ALS patients. (Nederlands Dagblad, 11-9-2009)
	The achievements of medicine are in many ways a blessing. The burden of pain, dyspnea and dehydration on the deathbed can be alleviated with palliative care and palliative sedation. (Trouw, 20-2-2010)
Suffering can be meaningful; A natural death is better than a non-natural death	Each of us has probably once experienced someone in the immediate environment who was dependent on us. This is not a purely negative experience. The physical proximity allows for intimacy and the relationship thereby becomes deeper. Let us cherish the memory of such experiences. It is possible that this reduces the need to control dying. It can help us in taking a relaxed view at the end of life. Just like chips and a plate of hot food, a death that is normal probably tastes best. (Trouw, 20-2-2010)
Society should protect the vulnerable; Human life should be preserved	[The Human Rights Commission of the United Nations] is most concerned, however, about Dutch euthanasia policy. Do vulnerable groups such as older people and the disabled not deserve to be better protected? […] The UN Committee defends the widely shared view that life is too precious to be terminated. Moreover, they correctly point to the fact that social perceptions of old age and frailty can also be influential. Where dependence is linked to dehumanization and indignity, the 'art of dying well' (the true meaning of euthanasia) disappears. (Nederlands Dagblad, 18-7-2009)
Performance of euthanasia is disturbing for the physician	Doctors are there to deal with medical affairs. They are there to keep people alive and to help them die in case of severe illness. But performing euthanasia is for almost every doctor highly personally invasive and stressful. (Volkskrant 20-3-2010)
Performance of euthanasia is disturbing for the physician; It is outside the scope of normal medical practice; Abrupt death (euthanasia) is an awful way of dying for relatives	The book (JR: “Verlossers Naast God” / Saviours besides God, by Anne Mei The) pains us, because it painfully shows what an enormous impact euthanasia can have on a doctor. 'My kids run to me and embrace me. I am fighting back my tears” is the last sentence of a doctor who comes home after he has “helped” a man. Doctors do not want to disappoint patients who trust them. But a request for euthanasia is not normal, just as the act itself is not. Even relatives are often shocked at their own reactions. Discontent among the "silent majority" of doctors began in 2001. The compassionate help provided in the past was exchanged for an abrupt action that goes against everything a doctor normally represents. (Nederlands Dagblad 2-12-2009)

The most frequently mentioned argument for euthanasia referred to the desirability of self-determination by the patient (40%) and to the contribution made by euthanasia to alleviating suffering (28%). Other arguments in support of euthanasia were that it contributes to a good and dignified death (11%), that people requesting euthanasia should not be abandoned (5%), and that euthanasia prevents horrible suicides (5%). The regulation of euthanasia was frequently referred to, predominantly claiming that regulation enhanced a careful and safe practice, and countering arguments against euthanasia suggesting it is a poorly practiced and difficult to regulate practice (20%). In three articles, euthanasia was argued to be a potential solution for the aging of the population or a means to reduce medical costs.

Arguments against euthanasia also often referred to the suffering of patients, predominantly by arguing that suffering should be alleviated by better care rather than euthanasia (36%). Further, it was argued that suffering can be meaningful in itself, and that suffering can mostly be prevented, for example by reducing overtreatment or resisting the excessive medicalisation of death, so that euthanasia would not be necessary (8%). Other arguments against euthanasia were that it is disturbing for the physicians who carry it out (13%) and that euthanasia is difficult to regulate (11%). Societal or religious aspects were also referred to as arguments against euthanasia. The most mentioned arguments in these categories were that society should protect the vulnerable (11%) and that human life in general should be protected (19%). Finally, some articles contained the argument that it is impossible to prevent misuse of euthanasia given the fact that the formal review of each case required in the Dutch regulatory system takes place only after the deed has been done (4%).

## Discussion

The legalisation of euthanasia and physician-assisted suicide in the Netherland is considered by many to be a social experiment [7], and people in many other countries keep a (sometimes critical) eye on Dutch practices and debates [8]. While several studies have provided reliable estimates about the frequency and characteristics of the practice of euthanasia in the Netherlands [9], this is the first study providing an overview of how euthanasia is described and debated in Dutch newspapers. Our study shows that nearly a quarter of Dutch newspaper articles used the term euthanasia for practices that were outside the scope of Dutch euthanasia law. Further, our analysis showed the wide-ranging nature of the public debate. Euthanasia was discussed in varying contexts and situations: for terminally ill patients but even more so for other groups such as older persons, persons with dementia, or persons with a psychiatric disorder. Also, there was a large variety of organisations and associations used as sources as well as there being variety in the arguments for or against euthanasia.

The term euthanasia, literally meaning a “good death”, has had different meanings depending on the historical and political context. Nowadays, euthanasia is understood by authorities to be the intentional ending of life by the administration of drugs by a physician at the explicit request of a patient[1], This definition is broadly accepted and adopted in legal regulations and in research. Yet nearly a quarter of the newspaper articles used the term euthanasia for practices that are outside the scope of this definition. In several instances, the term euthanasia referred to physician-assisted suicide (where the patient and not the physician administers the lethal drug), a practice that provokes quite similar moral debates as euthanasia, but is a different practice [1]. In other instances, the term euthanasia referred to practices that involve moral considerations that are different from those associated with euthanasia, such as the ending of life without a request of the patient (morally more problematic than euthanasia, many would feel) or the forgoing of potentially life prolonging treatments (usually morally less problematic, most would say). Also, the term was sometimes used for practices that do not involve a physician, including for example assisted suicide by non-physicians, suicide, or metaphorical usages. Taken together with the fact that 40% of the newspaper articles did not give any description of what practices the term euthanasia referred to, this means that one should be very cautious as to what people mean when they think they are discussing ‘euthanasia’. Because newspapers are generally considered to be an important source of health information [6], this kind of definitional variability is likely to feed misunderstanding and confusion in public debates. Moreover, for effective public health policy and compliance with Dutch legal regulations, a shared understanding of key concepts and terminology is essential.

In 2010, of all deaths in the Netherlands, 2.8% were the result of euthanasia [9]. Physician-assisted suicide was practiced less often, in 0.1% of all deaths. The large majority of euthanasia patients were diagnosed with cancer, were younger than 80 years of age, had a very short life expectancy and all were considered to be mentally competent when they made their request [9]. Euthanasia for persons who are old but not terminally ill, or who have (partly) lost mental capacity (e.g. due to a psychiatric disease, a coma or dementia) is practiced rarely. Yet euthanasia for these patients groups is quite commonly discussed in Dutch newspaper articles. Rather than reflecting practice, this reflects current medical and political debates about euthanasia which predominantly focus on the boundaries of current legislation. Examples of such boundary debates include the legitimacy of accepting “unbearable suffering” as a reason for euthanasia in patients with no serious medical condition [10,11], and the degree to which advance directives might substitute for a verbal request when the patient has become mentally incapacitated [12-14]. Media coverage of the practice of euthanasia, therefore, reflects a focus on more controversial cases rather than reporting on the majority of situations in which euthanasia is actually performed. While the media as such fulfils an important task of contributing to a societal debate and highlighting controversy, such coverage may lead to an inaccurate perception of the practice of euthanasia by the public [15].

Until recently, the Netherlands was known for tolerant policies on controversial issues such as abortion, drug abuse and euthanasia [16]. Factors often mentioned as contributing to these policies are the openness of the Dutch in discussing difficult issues, their tendency to prefer consensus and a strong trust in health-care providers [8,17]. This is also reflected in the fact that the Euthanasia Law receives ample support among the general public and health care professionals [18]. However, our study shows that Dutch public debate about euthanasia, especially its boundaries, does not necessarily involve an *easy* consensus. The opinions of both advocates and opponents allowing euthanasia for controversial groups are clearly visible, with a wide array of arguments for and against euthanasia being presented. The most commonly used arguments for euthanasia were that self-determination is a right and that the practice alleviates severe or unbearable suffering. The responsibility of society to regulate the response to the wish for assistance in dying of patients experiencing unbearable suffering was emphasised in these arguments, which are all central in current medical, ethical and political debates about euthanasia. A few newspaper articles argued that euthanasia might be a solution for the aging of the population or be a way of conserving scarce resources, which is obviously a morally more problematic argument, though perhaps attractive to journalists wanting to focus on more extreme positions. Arguments against the use of euthanasia refuted the right to self-determination and presented a different perspective on the core responsibilities of society, by focussing on the need to protect the vulnerable. There were also claims that suffering can be meaningful.

Our study also sheds light on a less debated aspect of the practice of euthanasia, that is, whether euthanasia contributes to a good death. This argument divides opponents and advocates of euthanasia. On the one hand, it is argued that euthanasia might contribute to a good, dignified, “civilized” death, for example by preventing distressing forms of suicide. On the other hand, euthanasia is described by some as too abrupt and unnatural for both patients and relatives and as a disturbing experience for physicians. This illustrates how perceptions about this practice and the dying phase in general may vary, and it underlines the need for medical practitioners to both counsel patients and their families about the course of their illness and dying phase, and to consider their own responses to such momentous end-of-life decisions as euthanasia.

## Conclusions

Nearly a quarter of the newspaper articles use the term euthanasia for practices that are outside the scope of Dutch euthanasia law, and newspapers typically discuss the more unusual cases such as euthanasia for patients with dementia or a psychiatric disorder. This has the potential to confuse the public debate about euthanasia, and means that physicians should be aware that their patients may have mistaken views about what euthanasia is, who it is for, and what circumstances lead to a decision to carry out euthanasia. Despite the legalisation of euthanasia in the Netherlands, the debate about its acceptability and boundaries is ongoing and both sides of the debate are clearly represented.

## Competing interests

The authors declare that they have no competing interests.

## Authors’ contributions

JACR and NJHR were responsible for the analysis of the data. All authors made substantial contributions to conception and design of the work, acquisition of data, or analysis and interpretation of data; drafting the article or revising it critically for important intellectual content; and final approval of the version to be published.

## Pre-publication history

The pre-publication history for this paper can be accessed here:

http://www.biomedcentral.com/1472-6939/14/11/prepub

## Supplementary Material

Additional file 1 Arguments for euthanasia in Dutch newspaper articles. Click here for file

Additional file 2 Arguments against euthanasia in Dutch newspaper articles. Click here for file
