# Supplementary material for: News media coverage of euthanasia: a content analysis of Dutch national newspapers
Source: BMC Med Ethics. 2013 Mar 6;14:11. doi: 10.1186/1472-6939-14-11 (PMC3599791; doi:10.1186/1472-6939-14-11)
Supplement: Additional file 1 — Arguments for euthanasia in Dutch newspaper articles. [file 1472-6939-14-11-S1.doc]

**Additional file 1 Arguments for euthanasia in Dutch newspaper articles***^a^*

|  | N=94  % |
| --- | --- |
| **Self-determination** | 40 |
| **Suffering** |  |
| It alleviates current suffering | 28 |
| It contributes to a good and dignified death | 11 |
| People should not be abandoned by denying them euthanasia | 5 |
| It prevents horrible suicides | 5 |
| Not all suffering can be alleviated by other means | 2 |
| It prevents future suffering | 2 |
| It prevents the medicalisation of dying | 1 |
| **Regulation** |  |
| Regulation enhances carefulness | 20 |
| (Citizens’desire for) euthanasia happens anyway so it might as well be regulated | 4 |
| The law says that anyone has a right to euthanasia | 2 |
| **Society** |  |
| It is a solution for aging of the population/ scarcity of resources / medical costs | 3 |
| It is a marker of a civilized society | 2 |
| It is what the majority of the society wants (democracy) | 1 |

a. More than one argument possible per article
