# Supplementary material for: News media coverage of euthanasia: a content analysis of Dutch national newspapers
Source: BMC Med Ethics. 2013 Mar 6;14:11. doi: 10.1186/1472-6939-14-11 (PMC3599791; doi:10.1186/1472-6939-14-11)
Supplement: Additional file 2 — Arguments against euthanasia in Dutch newspaper articles. [file 1472-6939-14-11-S2.doc]

**Additional file 2 Arguments against euthanasia in Dutch newspaper articles** *^a^*

|  | N=94  % |
| --- | --- |
| **Against self-determination** |  |
| God disposes of life and death | 7 |
| How voluntary is a ‘voluntary request’? | 2 |
| **Suffering** |  |
| Suffering can/should be alleviated by better care | 36 |
| Suffering can/should often be prevented | 8 |
| Suffering can be meaningful | 6 |
| Suffering is subjective and difficult to measure | 3 |
| Abrupt death (euthanasia) is an awful way of dying for patient or relatives | 2 |
| Suffering can pass | 1 |
| Unclear whether future suffering (as a reason for euthanasia) will occur | 1 |
| **Issues relating to regulation, practice or responsibilities** |  |
| Performance of euthanasia is disturbing for the physician | 13 |
| It is difficult to regulate | 11 |
| A physician should never decide about someone else’s quality of life | 5 |
| It is outside the scope of physicians’ responsibilities | 3 |
| It is a poorly performed practice (e.g. it is -against regulations- sometimes performed by nurses or second opinions are of insufficient quality) | 3 |
| It is outside the scope of normal medical practice | 2 |
| Suicide (without physician involvement) is better | 1 |
| **Society** |  |
| Society should protect the vulnerable | 11 |
| Social pressure on older or sick people burdens them with an ‘option’ | 5 |
| Regulating euthanasia is too individual an approach; more attention should be paid to the broader context (such as to why some people do not want to live anymore and the role of the social context) | 2 |
| The personal wish for a good death should not be made political | 1 |
| **Naturalness / non-interference with human life** |  |
| Human life in general should be preserved | 19 |
| A natural death is better than a non-natural death | 4 |
| It is a sign of disrespect for disabled people | 3 |
| It is murder | 1 |
| **Undesirable consequences** |  |
| Euthanasia is final, there is no way back | 5 |
| Impossible to prevent misuse when review takes place after performance | 4 |
| Provokes impulse decisions by patients | 2 |
| Leads eventually to less attention for palliative care | 1 |

a. More than one argument possible per article
